# Colleters, Extrafloral Nectaries, and Resin Glands Protect Buds and Young Leaves of *Ouratea castaneifolia* (DC.) Engl. (Ochnaceae)

**DOI:** 10.3390/plants10081680

**Published:** 2021-08-16

**Authors:** Elder A. S. Paiva, Gabriel A. Couy-Melo, Igor Ballego-Campos

**Affiliations:** Departamento de Botânica, Instituto de Ciências Biológicas, Universidade Federal de Minas Gerais, Belo Horizonte 31270-901, MG, Brazil; epaiva@icb.ufmg.br (E.A.S.P.); gabriel-couy@hotmail.com (G.A.C.-M.)

**Keywords:** calcium oxalate crystals, colleter, extrafloral nectaries, resin gland, bud protection, plant-environment interaction

## Abstract

Buds usually possess mechanical or chemical protection and may also have secretory structures. We discovered an intricate secretory system in *Ouratea castaneifolia* (Ochnaceae) related to the protection of buds and young leaves. We studied this system, focusing on the distribution, morphology, histochemistry, and ultrastructure of glands during sprouting. Samples of buds and leaves were processed following the usual procedures for light and electron microscopy. Overlapping bud scales protect dormant buds, and each young leaf is covered with a pair of stipules. Stipules and scales possess a resin gland, while the former also possess an extrafloral nectary. Despite their distinct secretions, these glands are similar and comprise secreting palisade epidermis. Young leaves also possess marginal colleters. All the studied glands shared some structural traits, including palisade secretory epidermis and the absence of stomata. Secretory activity is carried out by epidermal cells. Functionally, the activity of these glands is synchronous with the young and vulnerable stage of vegetative organs. This is the first report of colleters and resin glands for *O. castaneifolia*. We found evidence that these glands are correlated with protection against herbivores and/or abiotic agents during a developmental stage that precedes the establishment of mechanical defenses.

## 1. Introduction

The botanical family Ochnaceae has a pantropical distribution, comprising 27 genera and approximately 500 species [1], with its center of diversity being the Neotropics. Most of the diversity of Neotropical taxa is in the Amazon Basin, with just a few extra-Amazonian distributions restricted to Andean forests or the Brazilian Cerrado and Atlantic Forest [2]. The *Ouratea* genus, with about 300 species, is the largest and most diverse of the family [2]. The genus is widely distributed across several phytogeographic domains of Central and South America [3,4]. Many species have been recently described as *Ouratea*, demonstrating the limited study of the group and the cause of many taxonomic controversies (see [4]).

Secretion and secretory products seem to be important features for species of *Ouratea*. Representatives of this genus possess a pair of conspicuous stipules, in which there is, at least for some species, an extrafloral nectary (EFN) on the abaxial face [5]. Furthermore, species of *Ouratea* are a rich source of flavonoids and biflavonoids, and show potential as constituents of medicines; triterpenes, diterpenes, steroids, monosaccharides, and triacylglycerides are also common in this plant group [6].

Plant secretions are related to several forms of plant–environment interactions. Floral and vegetative buds constitute a vulnerable portion of plants and, thus, physical and chemical protections have often been found in these meristematic regions. Shoot buds usually possess substances produced by glands as distinct as colleters, nectaries, resin-producing glands, elaiophores, or secretory cavities [7]. 

The protective roles of some bud secretions have long been studied, as attested by the reports made by Groom [8], who stated that “*many buds have a great protective auxiliary in the secretion which covers and fills them. This secretion consists of gummy mucilage or resin, or both together; it is secreted by the general epidermis, by colleters, or by “leaf-teeth*”.” The substances present in bud secretions can protect vegetative and reproductive buds against several environmental stresses ([9,10] and references therein). These substances act complementary to the protection provided by enveloping shoot apical meristems by superimposing cataphylls and undeveloped leaves [11].

Damage to shoot apices presents a high cost because they are essential for plant growth [12]. Therefore, investments in protecting the apical meristem are advantageous, since the resprouting ability is a key functional trait that enables plant populations to persist after the destruction of living tissues from disturbance [13].

Knowledge regarding chemical defenses in shoot buds of Ochnaceae is far from comprehensive, although nectar secretion in scales and stipules of some taxa is well known. Many authors have recently reported the presence of glands in the family [14,15,16,17], sometimes relating them to bud protection [16]. Nevertheless, many aspects of secretion in the family are still poorly understood. 

Considering this knowledge gap, we investigated the anatomy, histochemistry, and ultrastructure of the colleters, nectaries, and resin glands present in the buds and developing leaves of *Ouratea castaneifolia* (DC.) Engl., an arboreal-shrub species of the Cerrado and Brazilian semi-deciduous forests [18]. This evergreen species possesses leaves with a long lifespan, which persist for more than two years [19]. We present novel anatomical and ultrastructural data regarding some glands of Ochnaceae, and further discuss functional aspects. 

## 2. Results

### 2.1. Bud Dynamics and Structural Aspects

**Figure 1:**
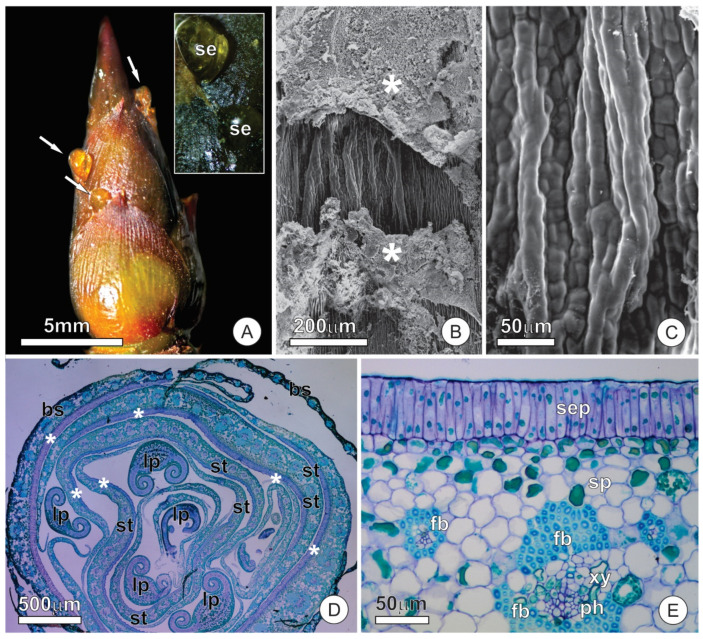
Resin glands of bud scales and stipules of *O. castaneifolia*: (**A**) Vegetative bud showing resin accumulation on the surface of bud scales (arrows). The insert shows released secretion towards the abaxial side of the bud scales; (**B**,**C**) The surface of resin-glands showing corrugated aspect and secretion residues (*). (**D**) Cross-section of a bud showing an overlapped arrangement of bud scales, stipules, and leaf primordia. Note the distinct epidermis at the abaxial face of bud scales and stipules (*); (**E**) Cross-section of a stipule showing adaxial secretory epidermis and the overall arrangement of the mesophyll and vascular tissue. Note the fiber cap that surrounds the vascular bundles; bs = bud scale, fb = fiber cap, lp = leaf primordium, ph = phloem, se = secretion, sep = secretory epidermis, st = stipule, xy = xylem.

Adult individuals of *O. castaneifolia* exhibit rhythmic growth, with one event of shoot growth per year and a long bud dormancy period lasting about ten months. During this stationary phase, the vegetative buds are protected by overlapping bud scales covering the vegetative apices (Figure 1A). The buds re-establish meristematic activity and start a new phase of vegetative growth before the end of the dry season (April–September), resulting in new leaves. After bud burst, shoot elongation occurs for about two months, when leaves grow, differentiate, and become strongly coriaceous and hard.

Flowering occurs at the end of leaf sprouting, and the vegetative buds go into dormancy, remaining in a resting state until the next annual vegetative cycle. The leaf primordia are produced at the beginning of the vegetative growth period and can be found at the apical portion of buds, each protected by a pair of stipules. The scales and stipules are foliaceous, wide, long (0.5 × 0.8 cm and 0.6 × 1.2 cm, respectively), and overlap the apical meristem (Figure 1A). The deciduous scales fall during bud burst, and stipules show abscission at the end of leaf blade expansion. Mature leaves of *O. castaneifolia* are simple, sclerophyllous, and coriaceous, with long marginal teeth resembling spines.

### 2.2. Resin Glands

The median-basal portion of the adaxial surface of bud scales and stipules shows secretory epidermis related to resin secretion. The gland presents an irregular outline, with variable size among the different scales and stipules, sometimes occupying more than half of the adaxial surface. The surface of the gland is corrugated, with a smooth cuticle, and often covered by secretion residues. Stomata, pores, or cuticular ruptures were not observed on the gland surface (Figure 1B,C). 

The resin gland possesses a secretory epidermis with columnar cells arranged in a palisade-like pattern (Figure 1D,E). The anticlinal surface of the secretory cells is about three times longer than the ordinary cells of the epidermis. The secretory cells have dense cytoplasm and large nuclei compared to other epidermal cells (Figure 1D). The mesophyll of scales and stipules have similar arrangements and cell types, being homogeneous and formed by round parenchyma cells (Figure 1D,E). Parenchyma cells of the portion of mesophyll underlying the gland do not have distinguishing characteristics (Figure 1D,E). The vascular bundles, arranged in the median portion of the mesophyll, are similar in scales and stipules; they are collateral and present an extensive cap of fibers on both abaxial and adaxial sides (Figure 1E). No change was observed in the vascular bundles towards the secretory region of the resin gland.

**Figure 2:**
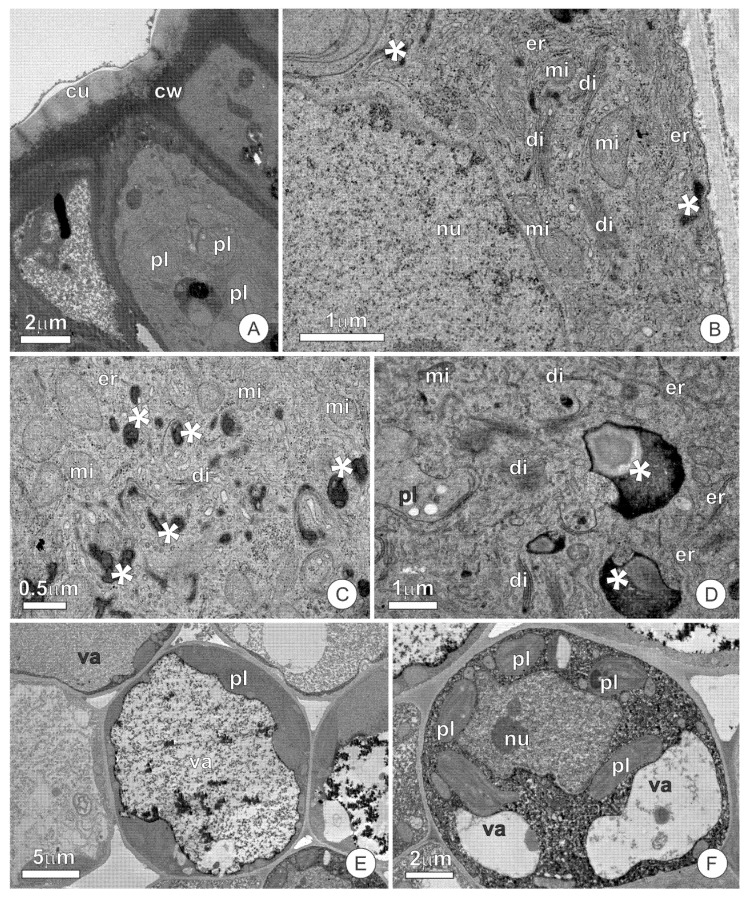
Ultrastructural aspects of the resin glands of *O. castaneifolia*: (**A**) Overall aspect of the secretory epidermis in cross-section. Note the dense protoplast of the cells. (**B**–**D**) Secretory cells showing organelle-rich protoplast with numerous mitochondria, dictyosomes, and segments of the smooth endoplasmic reticulum. Note the numerous deposits of osmiophilic secretion throughout the cytosol (*) (**E**,**F**). Cells of the subglandular parenchyma showing large vacuoles and phenolic contents. cu = cuticle, cw = cell wall, di = dictyosome, er = endoplasmic reticulum, mi = mitochondria, nu = nucleus, pl = plastid, va = vacuole.

The secretory cells have a dense protoplast (Figure 2A), with a conspicuous nucleus and numerous organelles (Figure 2B). Mitochondria, dictyosomes, and the endoplasmic reticulum are the most abundant organelles, appearing scattered throughout the cytoplasm (Figure 2B–D). The endoplasmic reticulum is predominantly smooth, mainly appearing parallel to the plasma membrane and forming an extensive network permeating the entire cytoplasm (Figure 2B,C). Plastids are scarce and possess dense stroma and poorly developed inner membranes. The vacuome of secretory cells is inconspicuous and limited to small vacuoles. The presence of secretion, forming deposits of varying volumes throughout the cytosol, is striking in these cells (Figure 2C,D). The secretion observed in the cytosol is heterogeneous, with a peripheral portion strongly osmiophilic and a central region with a granular aspect (Figure 2D). The most striking feature in the cells of subglandular parenchyma is the presence of a large central vacuole, which has phenolic content (Figure 2E). Plastids, mitochondria, and endoplasmic reticulum are the predominant organelles in the extravacuolar cytoplasm (Figure 2E,F).

**Figure 3:**
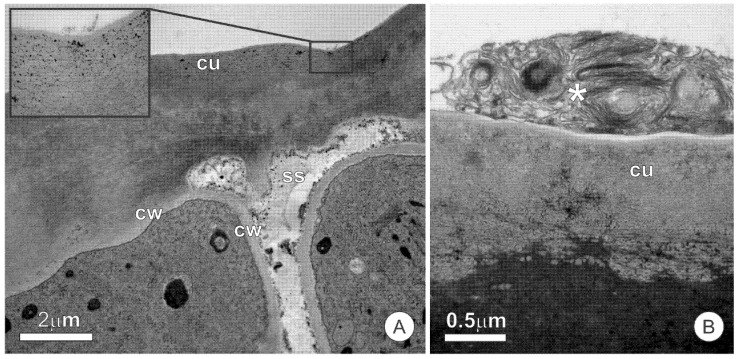
Cuticle structure in the secretory epidermis of resin glands: (**A**) Cross-section of the epidermic cells showing osmiophilic microdeposits inside the cuticle. A small subcuticular space can be observed. (**B**) Secretory residues are forming lamellar structures (*) on the surface of the cuticle. cu = cuticle, cw = cell wall, ss = subcuticular space.

The ultrastructural analysis of the cuticle did not detect channels or ruptures that allow for the release of the secretion. Microdeposits of osmiophilic material are observed in the cuticle, similar to that observed in the protoplast (Figure 3A). The formation of subcuticular spaces is rarely observed and is mainly limited to a few cells (Figure 3A). Secretion residues, which spread throughout the adaxial surface of scales and stipules and disperse throughout all the structures they encompass, are present on the cuticular surface, sometimes forming lamellar structures (Figure 3B).

### 2.3. Colleters

**Figure 4:**
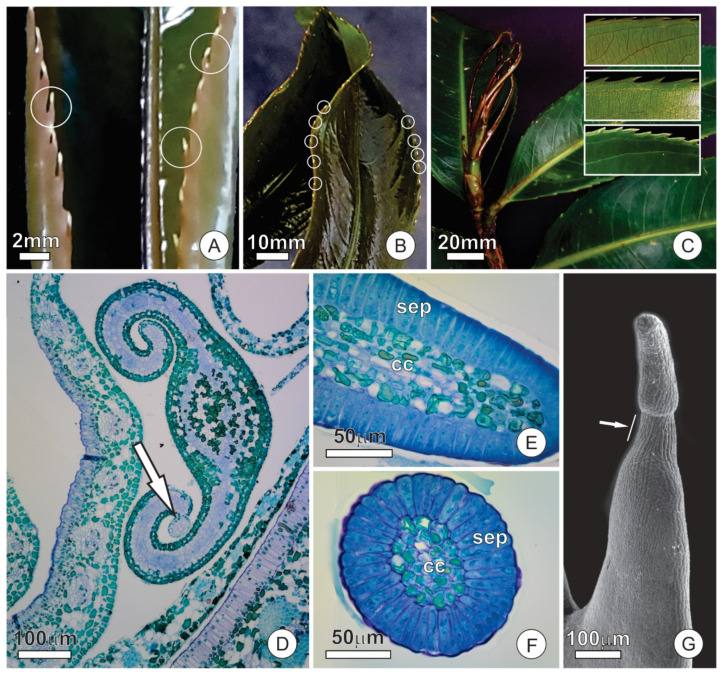
Distribution and structure of leaf colleters of *O. castaneifolia* leaves: (**A**,**B**) Young leaves, at the final stage of expansion, with hyaline colleters at the apex of the marginal teeth (circles). (**C**) Vegetative bud showing young leaves above mature leaves (from last sprouting event). Young leaves are shinny due to spread of colleter secretion; mature leaves present prominent marginal teeth. The inserts show the sequence of leaf maturation; notice the marginal teeth of the unexpanded young leaf (on top), followed by colleter abscission, intense sclerification and, finally, the fully-developed marginal spines (bottom). (**D**) Cross-section of a young leaf with involute ptyxis. Note the colleter at the leaf margin (arrow). (**E**) Longitudinal section of a colleter showing the secretory epidermis with columnar palisade cells and a central axis. (**F**) Cross-section of a colleter showing secretory epidermis surrounding the central axis. (**G**) Scanning electron micrograph of a marginal tooth in a young leaf. Note that the conical-shaped colleter at the apex is connected through a peduncle region (arrow). cc = central axis; sep = secretory epidermis.

In the early stages of leaf differentiation, the marginal teeth present a colleter at the apex (Figure 4A,B). The colleters are long (up to 1 mm-long), pedunculate, and hyaline (Figure 4B). The peduncle region becomes sclerified and constitutes the marginal spines on mature leaves, leaving no evidence of the previous existence of the colleters (Figure 4C).

The colleters are formed in the early stages of leaf blade differentiation and are functional in young, unexpanded leaves (Figure 4D). Colleters persist in secretory activity throughout leaf expansion, but become senescent, turn brownish, and detach from the leaf after this stage. The secretory portion of colleters is conical, with a stomata-free epidermis covered by a smooth cuticle (Figure 4E–G). The fully-developed colleters present a secretory epidermis with columnar palisade cells that surround a parenchymatous central axis (Figure 4E,F).

**Figure 5:**
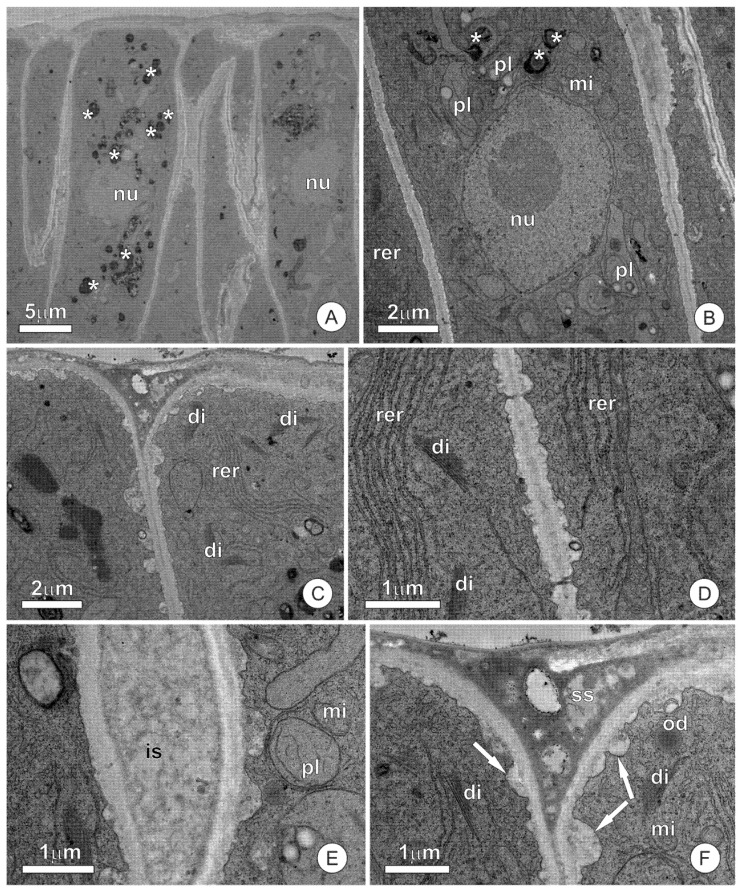
Ultrastructural aspects of the colleters of *O. castaneifolia*: (**A**) Overall aspect of the secretory epidermal cells showing dense protoplast and numerous osmiophilic inclusions (*); (**B**–**D**) Secretory cells showing large nuclei and organelle-rich cytoplasm with numerous mitochondria, plastids, and segments of the rough endoplasmic reticulum. Note oil droplets within the plastids and dispersed throughout the cytosol along with osmiophilic inclusions (*) in (**B**); (**E**,**F**) Details of the sinuous plasma membrane of secretory cells. Note the formation of irregular periplasmic spaces (arrows) and the accumulation of flocculated material within intercellular spaces and the subcuticular space. di = dictyosome, is = intercellular space, mi = mitochondria, nu = nucleus, od = oil droplet, pl = plastid, rer = rough endoplasmic reticulum, ss = subcuticular space.

In the secretory stage of colleters, the cells of the epidermal layer possess a dense and organelle-rich protoplast (Figure 5A–C). These cells display a large nuclei with uncondensed chromatin and conspicuous nucleoli (Figure 5B). The rough endoplasmic reticulum, mitochondria, and plastids complete the cytoplasmic organelles of the secretory cells (Figure 5B–D). Plastids have a poorly developed endomembrane system, and oil droplets that are similar to others observed free in the cytosol (Figure 5B). Dictyosomes are distributed throughout the cytoplasm, although they appear more numerous in the distal portion of cells (Figure 5C). The plasma membrane is sinuous, with the formation of irregular periplasmic spaces, within which the presence of amorphous and flocculated material can be observed (Figure 5C,E,F). This material is also accumulated in the intercellular spaces formed between palisade cells, in subcuticular spaces, and in large periplasmic spaces at the distal portion of the secretory cells (Figure 5E,F). The vacuome consists of small vacuoles, which are rare in most cells of the secretory epidermis. The parenchyma cells of the central axis present a set of organelles similar to those described for the secretory epidermis. However, these parenchyma cells possess few extravacuolar organelles due to the large central vacuoles filled with phenolics.

### 2.4. Extrafloral Nectaries

**Figure 6:**
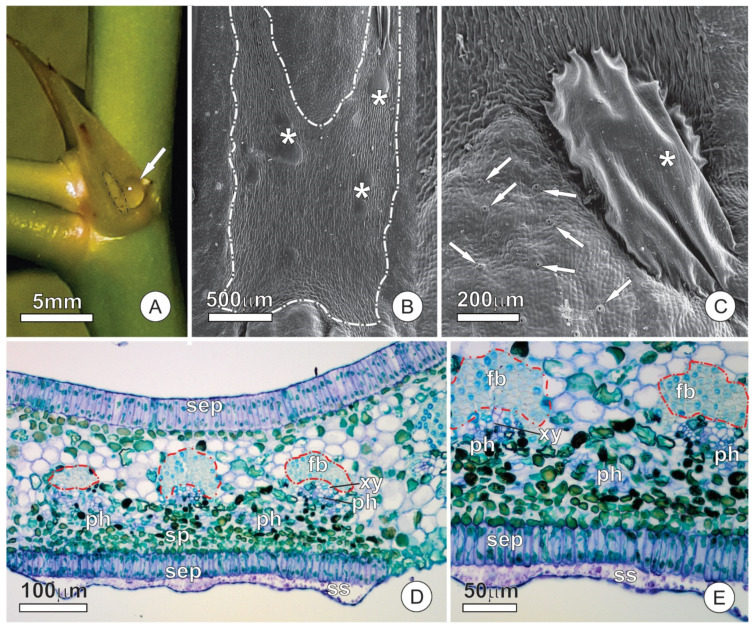
Structural aspects of extrafloral nectaries in stipules of *O. castaneifolia*: (**A**) Overview of a nectary (dashed line) in a stipule. Note the large nectar droplet (arrow). (**B**,**C**) Surface view of the nectary showing conspicuous subcuticular spaces (*) where nectar accumulates before being released. In (**C**), note the contrasting presence of stomata (arrows) on the ordinary surface of the stipule versus their absence over the nectary. (**D**) Cross-section of a stipule with a resin gland on the adaxial surface and a nectary on the abaxial surface. (**E**) Cross-section of a stipule showing the nectary portion. Note the dense arrangement of the subglandular parenchyma of the nectary and the presence of a large subcuticular space. A fiber cap (red dashed line), which is interrupted towards the nectary, surrounds the vascular bundles. fb = fiber cap, ph = phloem, sep = secretory epidermis, sp = subglandular parenchyma, ss = subcuticular space, xy = xylem.

A region of secretory cells stands out on the abaxial face of stipules (Figure 6A). This region is involved in the synthesis and release of nectar and constitutes an extrafloral nectary (EFN). The glandular surface is smaller than that of resin glands, slightly elongated in the axial direction, and also distinguished from the ordinary surface of the stipule by the absence of stomata, which are frequent in non-secretory portions (Figure 6A,B). Subcuticular spaces form conspicuous spaces on the secretory surface, which appear in different regions and reach large dimensions (Figure 6B,C), sometimes extending throughout the entire gland surface.

**Figure 7:**
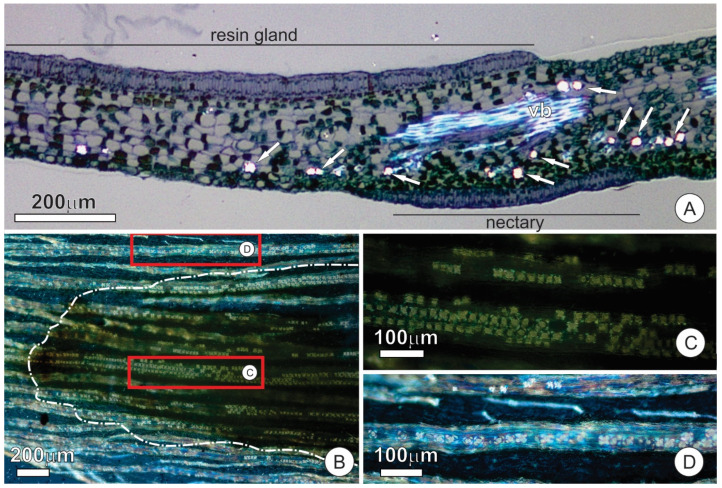
Distribution of calcium oxalate (CaOx) crystals in stipules of *O. castaneifolia*; all images were taken under polarized light: (**A**) Longitudinal section of a stipule showing greater accumulation of crystals (arrows) towards the abaxial surface and the nectary portion. Note that crystals are absent under the resin gland (adaxial face); (**B**–**D**) Surface view of the nectary portion (dashed line in (**B**)) showing the distribution of crystals. Note the numerous crystals in the nectary (**C**) in comparison to the area outside the nectary (**D**); The rectangles in (**B**) indicate the detailed areas in C and D. vb = vascular bundle.

The EFN consists of a uniseriate secretory epithelium, secretory parenchyma, and vascular tissues (Figure 6D). Epidermal cells are arranged in palisades similar to those described for the adaxial face (Figure 6E). The cells of the secretory parenchyma are smaller, and the cytoplasm is denser than the other components of the mesophyll (Figure 6D,E). Vascular bundles in the vicinity of the nectary possess a gap on the abaxial fiber cap such that phloem cells make contact with the secretory parenchyma (Figure 6D,E). The secretory parenchyma of the EFN shows a remarkable presence of calcium oxalate (CaOx) crystals in the form of druses (Figure 7A–C). CaOx crystals are characteristically associated with the vascular bundles in both scales and stipules, especially in the parenchyma cells associated with the abaxial surface at the fiber cap limit. However, the crystals are more numerous where the cap of fibers is interrupted in the nectary region than in other areas of the stipule (Figure 7B–D).

**Figure 8:**
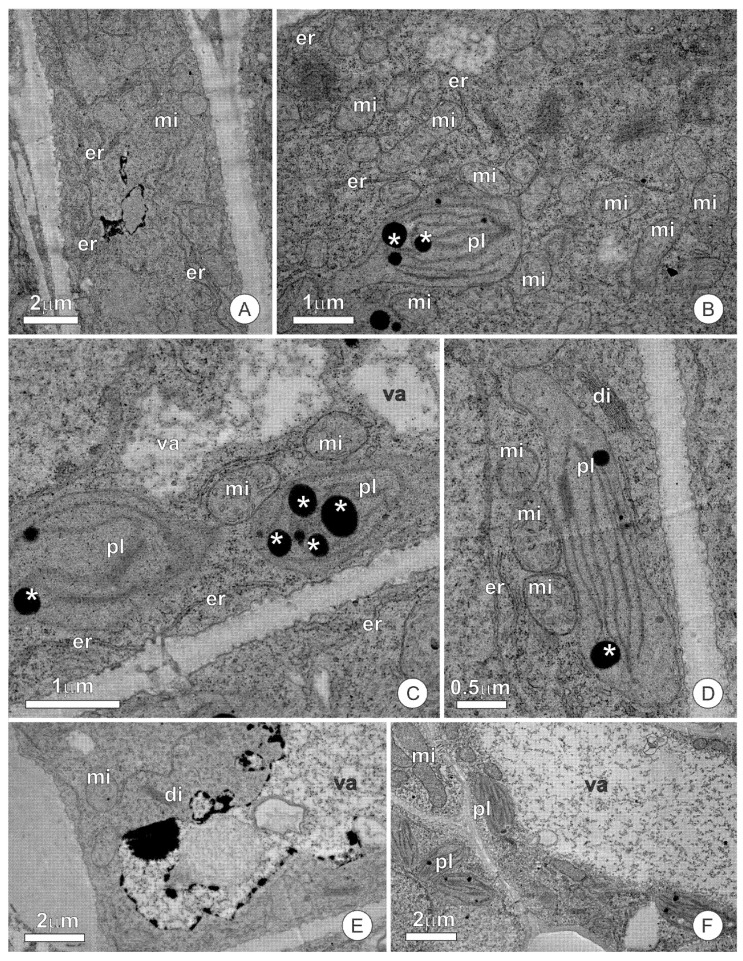
Ultrastructural aspects of the extrafloral nectaries of *O. castaneifolia*: (**A**) Overview of a secretory cell showing a dense protoplast; (**B**–**D**) Secretory cells showing organelle-rich cytoplasm with abundant mitochondria, segments of the rough endoplasmic reticulum, and plastids with a poorly developed inner membrane system. Note the numerous plastogobuli (*) dispersed in the stroma of the plastids and the small vacuoles filled with a flocculated content; (**E**,**F**) Cells of the subglandular parenchyma showing large vacuoles and extravacuolar cytoplasm rich in organelles. di = dictyosome, er = endoplasmic reticulum, mi = mitochondria, pl = plastid, va = vacuole.

The secretory cells present thin, pecto-cellulosic cell walls and cytoplasm rich in organelles, among which mitochondria, segments of the rough endoplasmic reticulum, dictyosomes, and plastids are the most representative (Figure 8A–D). Mitochondria have well-developed cristae and are distributed throughout the cytoplasm (Figure 8C,D). In the secretory stage, the dictyosomes appear inactive, with rare vesicles being produced (Figure 8D). Plastids have electron-lucent stroma, with a poorly developed inner membrane system with few grana thylakoids; plastoglobuli are dispersed in the stroma (Figure 8C,D), while starch is markedly absent. The few observed vacuoles are small and filled with a flocculated content (Figure 8C). Secretory cells of the epidermis were observed to connect via plasmodesmata (Figure 8C). Although secretory parenchyma cells have large vacuoles, the extravacuolar cytoplasm is organelle-rich and shows a composition similar to that of the cells of the secretory epidermis (Figure 8E,F).

### 2.5. Histochemistry and Sugar Analysis

Histochemical tests revealed a mixture of hydrophilic and lipophilic components, including terpenoids, mucilage, lipids, and proteins in both the resin-producing gland and colleters. Terpenoids were the most abundant and strongly marked by NADI reagent in the resin-producing glands, while mucilage was less conspicuous. Conversely, NADI reagent showed a weak reaction in the colleters, and both the protoplast and exudate marked strongly with Ruthenium Red. Differential coloration granted by the NADI reagent suggests that the terpene content is associated with essential oil production. Lipids and proteins were seen in both the protoplast and exudate of colleters but were absent in the resin-producing gland. 

The secretion exuded by the EFNs tested positive for glucose by glucose strip tests, indicating a sugary secretion and confirming nectar release. Tests with Xylidine Ponceau indicated the presence of structural proteins in the protoplast of nectary cells, but other tests yielded negative results. The results for all histochemical tests performed are summarized in Table 1.

## 3. Discussion

### 3.1. Anatomy

The secretory portion of the studied glands share some similarities, mainly because epidermal cells are directly involved in the secretory process in all of them. The prevalence of epidermis in secretory processes is common to many other secretory structures of eudicotyledons, including colleters, nectaries, elaiophores, and other glands throughout distinct taxa [20,21,22,23,24,25,26].

Resin production by a patch of differentiated epithelium, as observed in *O. castaneifolia*, is uncommon. These secretions are often associated with trichomes, colleters, ducts, or cavities [7]. Buds of *Populus* spp. (Salicaceae) possess a palisade-like epidermis in the adaxial side of the stipules that secretes resin [7,20], as described here for *O. castaneifolia*. However, in *Populus*, the secretory epithelium is not restricted to a specific area, extending towards the entire adaxial surface, which is heavily ridged [20].

Nonetheless, the similarities between the secretory system in buds of *O. castaneifolia* and *Populus* species are worth mentioning. Apart from the stipular resin glands, *Populus* also possess specialized leaf teeth with resin-secreting glands and extrafloral nectaries (or hydathodes [7]). Thus, the glandular apparatus of these taxa might constitute an interesting case of convergence regarding bud protection within the Malpighiales.

The presence of a central axis in the colleters of *O. castaneifolia* that is very distinct from the epithelial cells indicates a mixed origin of this structure, encompassing both the protoderm and ground meristem. Therefore, such colleters can be considered as the “standard-type”, following Thomas [21]. Standard colleters occur in several taxa of angiosperms, most notably the Rubiaceae and Apocynaceae [9,21,22,26]. Colleters or colleter-like glands (i.e., thick glandular hairs) have been reported in a few species of Ochnaceae, although usually associated with the inner base of stipules, sepals, or leaves [14,15,16]. Marginal glands, however, are commonly reported in *Sauvagesia* [15,27,28] and several additional genera of the subfamily Sauvagesioideae [14]. Recently, Rios et al. [17] also demonstrated marginal colleters in two species of *Luxemburgia*. Nonetheless, data on the anatomy, ultrastructure, and secretory activity of these structures is lacking, and the present description appears to be unprecedented, to the best of our knowledge.

The colleters of *O. castaneifolia* are very conspicuous due to their contrasting colors. However, these structures have not been described until now, and the reason seems to be the asynchrony between the phase in which they occur and that of interest for taxonomic studies. By the time most, if not all, *Ouratea* species bloom, the leaf is already wholly differentiated, and the colleters have already suffered abscission. Thus, in taxonomic analyses, which are mainly made of fertile material, colleters are not seen; this fact appears strikingly in the descriptions of new species, whose morphological descriptions are detailed yet do not register the presence of colleters. This gap in the reports of temporary secretory structures has also been reported for extrafloral nectaries [29]. Given that serrate leaves are a remarkable character for *Ouratea* [30], it seems reasonable to suppose that colleters, which occur at the apex of each marginal tooth, are a characteristic shared by several species of this genus. The report of marginal colleters in *Luxemburgia*, together with recent data showing a high correlation between leaf teeth and glands in eudicots [17], might corroborate this hypothesis.

Based on their structure, the nectaries of *O. castaneifolia* could be classified as embedded nectaries, i.e., totally embedded in tissues of other organs [31]. Nonetheless, they comprise slight specializations of the epidermis and subjacent tissue rather than conspicuous and distinct units enclosed in the mesophyll. The observed lack of bundle caps towards the nectary tissue is also noteworthy, as it exposes the phloem directly to the secretory parenchyma. While most vascularized nectaries rely on variable extensions of phloem, xylem, or both [31,32], the nectaries of *O. castaneifolia* are vascularized by direct contact with the vascular bundles. This, in turn, indicates the requirement of a steady and direct supply of pre-nectar solutions from phloem. Usually, extrafloral nectaries lack starch reserves [33], as we observed here. This remarkable absence of energetic reserves seems to reinforce the role of phloem as the source of pre-nectar.

### 3.2. Ultrastructure and Secretion Mechanism

The overall aspect of the protoplast, including dense cytoplasm, conspicuous nuclei, and numerous organelles, corroborates the secretory nature of the cells comprising the glands of *O. castaneifolia* [7,34,35]. Additionally, evidence of accumulated material (osmiophilic and granulated), either scattered throughout the cytoplasm or associated with vesicles and other organelles, corroborate an intense secretory process and a secretion of mixed nature, as also observed in histochemical tests.

The presence of abundant mitochondria observed in all studied glands likely reflects an intense metabolic activity with high-energy requirements [34,36], while other organelles are involved in specific types of secretory products [9,34,37]. In this sense, the presence of abundant active dictyosomes in the colleters and resin glands indicates polysaccharide synthesis related to mucilaginous secretory products, as commonly demonstrated in several glands secreting mucilage or mixed-secretions [7,9,37,38,39]. The presence of mucilaginous material, as revealed by histochemical tests, corroborates this view. However, in the resin glands, dictyosomes were also associated with osmiophilic material, indicating their involvement in resin synthesis. While this is less common, some authors have previously indicated the association of Golgi bodies with osmiophilic material in resin-secreting glands [40,41]. The osmiophilic nature and the positive reaction for terpenoids in histochemical tests suggest that this material comprises the terpene fraction of the secretion. Terpenoid synthesis in plants likely occurs at different cellular sites, so that a resinous substance might be composed of distinct portions produced after intercellular exchange between various compartments [7,42]. Plastids and the endoplasmic reticulum are usually the most common organelles associated with these type of secretions [7,9,37]; the abundant presence of these organelles in the colleters and resin glands of *O. castaneifolia* indicates that they are also involved in the resinous portion of the secretion. The presence of oil droplets and abundant, rough endoplasmic reticulum in colleters corroborates the occurrence of lipids and proteins in the secretion, as detected by histochemical tests.

In the case of the nectaries, the absence of osmiophilic inclusions, along with the inconspicuous activity of the Golgi apparatus and an abundance of endoplasmic reticulum, is congruent with nectar secretion. According to Fahn [7], the endoplasmic reticulum is the dominant organelle in nectar-secreting cells, and the dictyosomes might be less developed during the secretory stage.

The secretory route in the colleters and resin glands is delineated by the presence of secretion products (lipophilic, granular, and amorphous inclusions) dispersed throughout the cytosol, periplasmic spaces, and subcuticular spaces, and is also included in the cuticle. In this sense, secretions produced in the various organelles involved are transported throughout the cytosol, potentially fusing and agglomerating before liberation in the periplasmic spaces. After this point, the secretion crosses the cell walls, usually accumulating in intercellular spaces and small subcuticular spaces before reaching the surface of the glands. Accumulation in the periplasmic space and other extracellular spaces indicates that a pressure-based model of secretion release is involved [43]. The presence of osmiophilic droplets in the colleters and resin-secreting glands indicates lipophilic material and is a typical feature of resin-secreting glands [7,44].

The ultrastructure of the secretory cells in the nectaries indicate a granulocrine secretion [7,34], in which the incoming pre-nectar is processed, transported in vesicles, and eliminated via fusion or invagination of the plasmalemma. The conspicuous subcuticular spaces observed in the nectaries of *O. castaneifolia* suggest cuticle rupture and nectar release in a cycling manner. This mechanism of nectar release is a common feature among stomata-free nectaries, in which nectar can be released by repetitive cycles of cuticle detachments and rupture [45].

### 3.3. Functional Aspects

Secretions, such as nectar, resins, and mucilages, associated with EFNs, resin glands, and colleters, respectively, are recognized for mediating plant–environment interactions. The resin-producing glands of *O*. *castaneifolia* are related to the protection of the bud itself, including the promeristem and all of the developing organs that it contains. In turn, the EFNs and colleters are related to protecting specific young organs, namely the developing leaves. The secretion observed in resin glands, in which we found essential oils in association with polysaccharides, is similar to those commonly observed in colleters, as these structures also show mixed secretions with both hydrophilic and hydrophobic compounds. Therefore, resin glands act in the protection of buds in a similar way that typical colleters do, both providing a coverage of secretion that might protect against biotic and abiotic factors. In fact, from a functional point of view, these structures can be considered analogous. Although there are controversies about the definition of colleters, the functional aspect seems to be preponderant for recognizing these structures [46,47]. While the scales and stipules of *O*. *castaneifolia* have resin glands formed essentially by a secretory epithelium, Reinales and Parra-O [16] described the presence of standard colleters in scales and stipules for the clade comprising *Rhytidanthera*, *Godoya*, *Cespedesia*, and *Krukoviella.* It is important to note that these colleters and resin glands have similar secretory activity and, most likely, perform the same function. The involvement of colleters in the protection of buds, especially those associated with stipules and scales, has been reported for several taxa [48]. Therefore, the evolution of the glandular system in vegetative buds of Ochnaceae proves to be an open and intriguing question.

The type of ptyxis showed by *O*. *castaneifolia*, and the arrangement of colleters at the leaf blade margin, seem to act in facilitating the spread of secretion throughout the leaf surface, on both sides, as suggested by Paiva [49]. Thus, these colleters seem to have an action directed at leaf blade protection. On the other hand, the meristem and young leaves in the phase that precedes the formation of colleters, are protected by the secretion of resin glands. In this way, there is no overlapping of functions but a complementarity between these two secretory structures.

There seems to be a correlation between the composition of the secretion of colleters and environmental factors. Tresmondi et al. [9] compared colleters of species from savanna environments with those from the forest and observed that resinous secretions prevail in the savanna environment, which is subject to greater luminous and water stresses. Considering that *O*. *castaneifolia* inhabits savanna (Brazilian Cerrado) and forest-edge environments, the presence of mixed secretion, both in the colleters and in the resin gland, seems to reflect a greater protection against desiccation.

Concerning mucilaginous secretions, such as that produced by colleters, Groom [8] argued that “hygroscopic substance like mucilage (and tannin) is an admirable means of controlling the water-supply of an organ for two reasons: first, the osmotic power of a solution increases with a rise of temperature; secondly, the osmotic power increases with the concentration of the solution. The result is that when a bud is in greatest danger of losing all its water—i.e., when the temperature is high and a considerable amount of water has been evaporated from the mucilage—the remaining water is held most firmly or a first supply of water is absorbed most fiercely”. Similarly, resins are also likely to reduce water loss by cuticular transpiration or even reduce leaf temperature by increasing radiation reflectance in hot, arid conditions [7,50]. This protection against water loss is even more critical in young organs because their cuticle and vascular tissues are incipient, compromising adequate transport and water retention (see [49]). Additionally, due to its chemical composition, lipophilic substance such as essential oils and oleoresins are frequently associated with protection against pathogens and herbivores [7,23].

The occurrence of EFNs was reported for eight species of *Ouratea* [5], including *O*. *castaneifolia* [23,51,52]. In these reports, the location of the nectaries is the same, that is, on the abaxial face of the stipules or cataphylls. Thus, in all species of *Ouratea* with reports of EFNs, these structures are ephemeral and seem to be related exclusively to the protection of young organs, given the caducous nature of the stipules to which they are associated. According to Machado et al. [23], the EFNs of species of *Ouratea* effectively protect plants against herbivores; EFNs of *O*. *spectabilis* are visited by several ant species that significantly reduce damage by lepidopteran caterpillars.

In the studied EFNs of *O*. *castaneifolia*, the highest concentration of calcium oxalate crystals coincides with the vascularized portion of these structures. The presence of these crystals is associated with the control of cytosolic calcium levels [53], which seems to be an essential factor for nectar secretion [54]. It is not by chance that the presence of these crystals is frequently reported in the nectaries of different plant taxa [23,55,56,57,58,59,60,61]. Although the presence of these crystals is often linked to some protection against the action of herbivores [58], in *O*. *castaneifolia*, and in most of the taxa in which they occur, this seems unlikely. It is important to emphasize that the crystals occur in the deepest layers of the nectary, leaving the cells with dense protoplast, which are more nutritious and vulnerable to herbivory, exposed towards the gland surface.

## 4. Materials and Methods

### 4.1. Plant Material

Plant material was collected from three adult individuals of *O. castaneifolia* growing on the Campus of the Universidade Federal de Minas Gerais, Belo Horizonte (Brazil). The plants were observed and sampled during the years 2019 and 2020. Whole vegetative buds and the median portion of several isolated bud scales, stipules, and young developing leaves were obtained from each of these individuals and subjected to the procedures below. For each of the portions obtained, all individuals were sampled in each of the procedures, with at least three replicates per individual.

### 4.2. Light Microscopy

For microscopy analysis, whole buds and samples of bud scales, stipules, and young leaves were vacuum infiltrated with Karnovsky’s fixative (paraformaldehyde 4% and glutaraldehyde 5% in phosphate buffer 0.1 M, pH 7.2; modified from [62]) for 5 min and left to set for 24 h in the same solution. Soon after, they were dehydrated in an increasing ethanol series (10–98%) and embedded in (2-hidroxiethyl)-methacrylate (Historesin embedding kit, Leica, Heidelberg, Germany). Transverse and longitudinal sections of the entire apex and fragments of stipules, bud scales, and young leaves were obtained using a rotary microtome (Hyrax M40, Carl Zeiss Mikroskopie, Jena, Germany). The 5–6 µm thick sections were mounted on glass slides and stained with toluidine blue (0.5% in acetate buffer 0.1 M, pH 4.7; modified from [63]). Analysis and image capture were performed using a light microscope (CX41RF, Olympus Scientific Solutions, Waltham, MA, USA) coupled to a digital camera (U-TV0.5XC-3, Olympus Scientific Solutions, Waltham, MA, USA) and a computer with an imaging software (LCmicro, Olympus Soft Imaging Solutions, Waltham, MA, USA).

### 4.3. Electron Microscopy

For scanning electron microscopy (SEM), whole buds and samples of bud scales, stipules, and young leaves were fixed in Karnovsky solution (paraformaldehyde 4% and glutaraldehyde 5% in phosphate buffer 0.1 M, pH 7.2; modified from [62]). Samples were left under vacuum for 5 min to improve infiltration, after which they were kept in the fixative for 24 h. The samples were then dehydrated in an increasing ethanol series (5–100%), critical-point dried (CPD030, Bal-Tec/Leica, Balzers, Liechtenstein), and coated with a palladium-gold alloy (MD20, Bal-Tec, Balzers, Liechtenstein). The samples were analyzed using a Quanta 200 scanning electron microscope (FEI Co., Eindhoven, The Netherlands).

For transmission electron microscopy (TEM), samples of bud scales, stipules, and young leaves were prepared to isolate fragments (1 × 1 mm) containing portions of the secretory glands. These samples were fixed in Karnovsky solution (paraformaldehyde 4% and glutaraldehyde 5% in 0.1 M phosphate buffer, pH 7.2; modified from [62]), infiltrated under vacuum for 5 min, and left in this fixative for 24 h. The fixed material was post-fixed in osmium tetroxide (1% in phosphate buffer 0.1 M, pH 7.2) for 2 h, dehydrated in an acetone series (30, 50, 70, 95, 100%), and embedded in low viscosity epoxy resin [64]. The material was then sectioned with an ultramicrotome (UC6, Leica, Deer-field, IL, USA) coupled with a diamond blade. The ultrathin sections (40–60 nm thick) were contrasted using a saturated solution of uranyl acetate and lead citrate [65]. The analysis was performed using a Tecnai G2-Spirit transmission electron microscope (Philips/FEI Co., Eindhoven, The Netherlands) at 80 Kv.

### 4.4. Histochemistry

Freshly collected samples of *O. castaneifolia* were used for histochemical tests. For each studied gland, samples were free-hand sectioned, subjected to histochemical tests, and mounted on glass slides. Sudan Red B (0.5%, in ethanol 95% and glycerin 1:1) was used for lipids (modified from [66]), Ruthenium Red (0.002%, aqueous solution) for mucilages [67], NADI reagent for oleoresins and essential oils [68], and Xylidine Ponceau (0.1% in acetic acid 3%) for proteins [69]. After treatment, the sections were briefly washed in the respective solvent of each test, and then finally washed in distilled water (for NADI test we used phosphate buffer 0.1 M, pH 7.2). An analysis was performed at the end of each test using a light microscope (CX41RF, Olympus Scientific Solutions, Waltham, MA, USA) coupled to a digital camera (U-TV0.5XC-3, Olympus Scientific Solutions, Waltham, MA, USA) and a computer with an imaging software (LCmicro, Olympus Soft Imaging Solutions, Waltham, MA, USA). Additionally, glucose strip tests (Alamar Tecno Científica, São Paulo, Brazil) were used to confirm the presence of sugars in the nectary secretion.

## 5. Conclusions

The vegetative buds of *O. castaneifolia* display a diverse secretory system comprised of resin-secreting glands, colleters, and extrafloral nectaries. There is marked synchrony of the secretory activity of these glands with the differentiation and expansion of young organs. Thus, it seems reasonable to assume that the secretory activity in these cases is correlated with the protection against herbivores and/or abiotic agents, since buds and young organs are vulnerable. Vegetative buds are vulnerable structures that have a high fitness value and are usually strongly defended. In *O. castaneifolia*, the defense system is expressed through mediators of plant–environment interactions, which prevail in young organs and act in a phase that precedes the development of mechanical defenses.

## Figures and Tables

**Table 1:** Results for histochemical tests performed in the glands of *O. castaneifolia* buds and young leaves.

Test	Target Substance	Resin Gland		Colleter		Nectary	
		Protoplast	Secretion	Protoplast	Secretion	Protoplast	Secretion
NADI reagent	Terpenoids (essential oils)	+	+	−	−	−	−
Ruthenium Red	Mucilage	−	−	+	+	−	−
Sudan Red B	Lipids	−	−	+	+	−	−
Xylidine Ponceau	Proteins	−	−	+	+	+	−
Glucose strip-test	Sugars (glucose)	N/A	N/A	N/A	N/A	N/A	+

+ positive, − negative, or weak reaction, N/A = not applicable.

## Data Availability

Not applicable.
